# E-Learning Satisfaction and Barriers in Unprepared and Resource-Limited Systems During the COVID-19 Pandemic

**DOI:** 10.7759/cureus.24969

**Published:** 2022-05-13

**Authors:** Taqi Mohammed Jwad Taher, Rami Bahaa Saadi, Ranya Riyadh Oraibi, Hasanain Faisal Ghazi, Sahar Abdul-Rasool, Faiz Tuma

**Affiliations:** 1 Family and Community Medicine, College of Medicine, Wasit University/College of Medicine, Wasit, IRQ; 2 College of Medicine, Wasit University, Wasit, IRQ; 3 College of Nursing, Al-Bayan University, Baghdad, IRQ; 4 Department of Medical Biosciences, University of the Western Cape, Western Cape, ZAF; 5 Surgery, Central Michigan University College of Medicine, Saginaw, USA; 6 Surgery, Western Michigan University Homer Stryker M.D. School of Medicine, Kalamazoo, USA

**Keywords:** distant learning., blended learning, barriers, student satisfaction, e-learning, covid-19

## Abstract

Background

The sudden and quick propagation of coronavirus-19 disease (COVID-19) has disrupted face-to-face lectures and practical sessions at Iraqi universities. E-learning has surfaced in most countries as an alternative way to continue educational programs. This study aimed to determine the degree of satisfaction and perceived barriers among college students with E-learning.

Methods

Students of two Iraqi universities studying through an online platform participated in this cross-sectional study. An online survey questionnaire was used to assess student perceptions of the level of satisfaction with and barriers to E-learning. Participants' non-identifying demographics were also collected.

Results

The majority of students (70.9%) were females, and more than half (57.9%) were from the Faculty of Science. About 64.8% of the students were not satisfied with the E-learning experience. Only 35.5% of the students attended synchronous electronic classes while the rest used asynchronous learning activities. Students’ level of satisfaction was poor, as only 6.4% of students strongly believed that tutoring was informative and that technology and educational technology were adequate. On the contrary, 69% of students strongly agreed that E-learning saved them time and money. Barriers that were perceived by the student were slow internet speed, power interruption, and the lack of face-to-face interaction.

Conclusions

E-learning has significant barriers that require investment in infrastructures and teaching skills development to make students learning satisfactory.

## Introduction

The unprecedented outbreak of coronavirus disease 2019 (COVID-19) has disrupted ‎education worldwide [1]. As a result, all educational institutions were forced to close [2], a step that has led to a drastic and complete switching to the electronic learning (E-learning) mode to stop the COVID-19 from spreading [3].‎‏ ‏Globally, an estimated two ‎billion students have been impacted by school delays since the beginning of the COVID-19 ‎pandemic [4]. To maintain the educational process's sustainability, E-learning has surfaced ‎as a convenient platform for learning [5-6]. ‎

The term E-learning is used here to indicate distance learning that uses online courses and materials through electronic devices and is hence called E-learning. E-learning has several advantages as well as disadvantages. Some of its advantages include strengthening student centricity, providing more ‎flexibility [7], and increasing student interaction through ‎asynchronous and synchronous resources [8-9]. Moreover, the E-learning platform offers ‎learners content and time control; thus, serving the learning goals and learner's ‎needs in a more advantageous manner [10]. Furthermore, it offers ‎swift accessibility since it is web-based, and once the content is published, users can ‎access it at any time and anywhere, provided that internet access is available [11].‎ Navarro and Shoemaker have shown that students who used E-learning were able to assimilate skills perhaps ‎better than students who studied traditionally [12]. E-learning was also effective in the case of ‎students who are quiet, easily intimidated, and slow learners who are unable to speak up and ‎show themselves in a classroom environment [13].‎

On the other hand, several drawbacks, such as lack of interest, delayed feedback or encouragement, or feelings of loneliness due to the lack of physical presence of classmates have been reported with E-learning systems [14]. Therefore, both tutors and students came across many ‎challenges, and universities are facing challenges in keeping course content consistent and valid ‎‎[8]. ‎Hence, further studies are needed to identify the specifics that enhance the advantages of E-learning, especially in systems with limited educational experiences in this field where E-learning is still in its infancy [15]. In Iraq, alongside conventional classrooms, many public universities have ‎launched restricted attempts to use the E-learning offering most of their learning services ‎online during the COVID-19 pandemic, including lectures and various evaluations across ‎multiple platforms. The aim of this study is to evaluate the satisfaction of college students using ‎E-learning and to investigate the perceived barriers that affect the ability to deliver online courses.‎

## Materials and methods

 A structured questionnaire was prepared to include four main sections. Section 1 comprised socio-demographic data. Section 2 was related to the facilities, devices, Internet network, E-learning program, and the learning system of the college. Section 3 included students’ perspectives of tutor performance and college support. Section 4 comprised questions regarding barriers experienced by students. With a few minor modifications, the questionnaire is borrowed from the previous findings by Ibrahim NK [16]. Sections 3 and 4 of the questionnaires were analyzed using the 5-point Likert scale. The Medical Council of Wasit University/College of Medicine deemed the study exempt from ethical approval for the anonymity of participants.

This questionnaire was piloted with 20 randomly selected students who were excluded from the final study to enhance questionnaire clarity. The questionnaire was sent out to all undergraduate students of the Humanity and the Science colleges of the two largest Iraqi universities, AL-Iraqia University and Wasit University (studies ending up with bachelor's degrees). First and second-year students were excluded, as they did not have any on-campus learning experience. Data were collected from ‎‎the 5th to the 20th of January 2021.

Statistical analysis

Data were analyzed using the SPSS software program version 26 released in 2019 by IBM Corp, Armonk, NY. Descriptive statistics were done using frequency and percentage for categorical data and using mean ± standard deviation for quantitative data. The participants' satisfaction level was divided into ‘satisfied’ and ‘not satisfied’ by the cutoff point mean of 3.40 (the mean above 3.40 is corresponding to the agree and strongly agree responses), and this is based on the mean of the 5-point Likert scale system regarding tutor quality, perceived usefulness, and facilitating condition. The chi-square test was used to assess any association between a categorical variable, and multiple linear regression was done to know the predictors for the satisfaction and barriers. A P-value equal to or less than 0.05 was considered significant.

## Results

Out of a total number of 870 students who were invited to participate in this survey, 800 students (91.95%) responded and were included in the analysis. The mean age of the participants was 22.05±1.95 years. In Table 1, the ‎females represented (70.9%) of the participants. More than half of the participants ‎‎473 (59.1%) live in urban and city centers. Three-hundred fifty-five (44.4%) participants were from families with a monthly salary between 500 and 1-million Iraqi Dinars (400-800 American USD).‎

**Table 1:** Sociodemographic features of the participants in this study

Socio-demographic variables		Frequency	Percentage
Gender	Male	233	29.1
	Female	567	70.9
Place of living	Cities	473	59.1
	Districts and sub-districts	264	33.0
	Villages and peripheries	63	7.9
Monthly family salary in Iraqi Dinar	Less than 500,000	245	30.6
	Between 500,000 – 1,000,000	355	44.4
	More than 1,000,000	200	25.0
College type	Science Colleges	463	57.9
	Humanity Colleges	337	42.1
College stage	Third stage	331	41.4
	Fourth stage	351	43.9
	Fifth stage	72	9.0
	Sixth stage	46	5.8
Preferable type of electronic learning	Synchronized learning	162	20.3
	Non-synchronized learning	142	17.8
	Blended learning	324	40.5
	Flipped learning	172	21.5
The device used for electronic learning	iPad	143	14.7
	Cellphone	701	72.0
	Computer	129	13.3
Internet source	Mobile 3G	162	18.2
	Home Wi-Fi	726	81.8
Platform used to access E-learning	Google Meet	526	35.5
	Google Classroom	466	31.5
	Free Conference Call	213	14.4
	Zoom	183	12.4
	Telegram	24	1.6
	Moodle	21	1.4
	Edmodo	20	1.4
	Go to meeting	12	0.8
	Skype	6	0.4
	Messenger Room	5	0.3
	Microsoft Teams	4	0.3

More than half of the participants (57.9%) were studying at the college of science. The majority of the students (726; 81.8%) used ‎home Wi-Fi to access the internet and attend E-classes (Table 1). Most students (72%) used ‎cell phones for electronic learning. Others used iPad (14.7%) and computers (‎‎13.3%), respectively. Online attendance (synchronous learning) was low, as only 35.5% of students indicated having attended E-classes by Google Meet followed by 31.5% for Google Classroom.

Students' degree of agreement with the satisfaction criteria varied (Table 2). Only 118 students (14.8%) strongly agreed that ‎the tutor properly and accurately committed to the course timetable and the planned time. ‎Furthermore, 115 students (14.4%) believed that the tutors were patient when they ‎interacted with the students and the E-class, whereas 51 students (6.4%) strongly believed ‎that their tutors were knowledgeable in Information and Communication Technologies.‎

**Table 2:** Frequency distribution of the satisfaction criteria of students

Satisfaction criteria	Strongly agree	Agree	Neutral	Disagree	Strongly disagree
	No. (%)	No. (%)	No. (%)	No. (%)	No. (%)
Tutor quality	Mean ± standard deviation			(3.07±0.82)	
The tutor could explain the concepts clearly through e-learning.	82 (10.3)	170 (21.3)	236 (29.5)	189 (23.6)	123 (15.4)
My tutor was knowledgeable in Information and Communication Technologies.	51 (6.4)	209 (26.1)	242 (30.3)	222 (27.8)	76 (9.5)
My tutor was patient when they interacted with me and the class on E-learning.	115 (14.4)	333 (41.6)	194 (24.3)	97 (12.1)	61 (7.6)
The group sessions were well-facilitated.	68 (8.5)	248 (31.0)	258 (32.3)	167 (20.9)	59 (7.4)
My tutor depends on interactive lectures to draw students' attention.	69 (8.6)	269 (33.6)	211 (26.4)	167 (20.9)	84 (10.5)
The tutor used adequate supportive methods for delivering lectures (Presentations, YouTube, pre-recorded videos, etc.).	104 (13.0)	314 (39.3)	159 (19.9)	139 (17.4)	84 (10.5)
The tutor committed to the course timetable and the planned time accurately.	118 (14.8)	253 (31.6)	166 (20.8)	143 (17.9)	120 (15.0)
The tutor chooses the most suitable time for the lectures that accommodate the students’ needs.	104 (13.0)	256 (32.0)	164 (20.5)	165 (20.6)	111 (13.9)
The tutor can give enough attention to every single student that needs it.	59 (7.4)	162 (20.3)	238 (29.8)	197 (24.6)	144 (18.0)
Perceived usefulness	Mean ± standard deviation			(3.12±1.06)	
E-learning prepares me well for doing exams without the need for On-Campus learning.	144 (18.0)	144 (18.0)	109 (13.6)	181 (22.6)	222 (27.8)
I can understand the subjects without the need for external resources.	92 (11.5)	176 (22.0)	134 (16.8)	220 (27.5)	178 (22.3)
E-learning developed my experience regarding the use of the new technologies of smartphones, apps, and using the internet more efficiently.	179 (22.4)	330 (41.3)	136 (17.0)	83 (10.4)	72 (9.0)
E-learning made it easy for me to access lectures than before.	165 (20.6)	245 (30.6)	143 (17.9)	144 (18.0)	103 (12.9)
E-learning saved me more time and money than before.	276 (34.5)	276 (34.5)	84 (10.5)	78 (9.8)	86 (10.8)
The student rating is fairer with E-learning than it was with On-Campus learning.	105 (13.1)	127 (15.9)	141 (17.6)	191 (23.9)	236 (29.5)
E-learning is substituting the classic learning during the COVID-19 pandemic lockdown.	145 (18.1)	268 (33.5)	143 (17.9)	113 (14.1)	131 (16.4)
Facilitating condition	Mean± standard deviation			(3.03±0.94)	
There is enough information and instruction provided from the college regarding E-learning and the programs used.	77 (9.6)	358 (44.8)	172 (21.5)	111 (13.9)	82 (10.3)
There is a specialist department of E-learning with enough experience in the college.	79 (9.9)	262 (32.8)	216 (27.0)	142 (17.8)	101 (12.6)
When I need help, the college or the specialist department of E-learning will be available on need (or as soon as possible).	95 (11.9)	294 (36.8)	156 (19.5)	147 (18.4)	108 (13.5)
The college provides enough supporting technical materials (paid subscriptions for known educational websites, apps, and official E-Mails).	72 (9.0)	175 (21.9)	152 (19.0)	188 (23.5)	213 (26.6)
Our college benefits from distant learning opportunities for addressing and controlling large numbers of students.	95 (11.9)	220 (27.5)	226 (28.2)	154 (19.3)	105 (13.1)
The college and the staff were supportive and motivated for distance learning.	81 (10.1)	194 (24.3)	272 (34.0)	143 (17.9)	110 (13.8)

The majority of students (552, 69%) perceived E-learning as useful and ‎strongly agreed that they are saving more time and money than before. Some students (92, 11.5%) ‎strongly agree that they can understand the subjects without the need for external resources. ‎However, about a quarter of the students (213, 26.6%) strongly believed that colleges did not provide enough supporting technical materials. Furthermore, 110 students (13.8%) strongly ‎disagree that the college staff were supportive and motivated for distance learning.

The perceived barriers to E-learning were variable (Table 3). The most common barrier was limited resources (Internet access and electrical power) (n=650) with a mean of 4.28±1.00, followed by the unsuitability of some ‎disciplines or the contents for E-learning such as clinical teaching (4.04±1.09). The mean for total barriers was 3.53±0.82 while the mean for total satisfaction (tutor quality, perceived usefulness, and facilitating condition) was 3.07±0.83 ‎‎(Minimum mean=1 and the maximum=5). ‎About two-thirds (n=518; 64.75%) of the students were not satisfied with the E-learning provided by their colleges, whereas 282 (35.25%) were satisfied.

**Table 3:** Frequency distribution of the perceived barriers items among students

Perceived barriers	Strongly agree No. (%)	Agree No. (%)	Neutral No. (%)	Disagree No. (%)	Strongly disagree No. (%)	Mean ±SD
My inadequate computer skills are a barrier to me.	98 (12.3)	206 (25.8)	137 (17.1)	237 (29.6)	122 (15.3)	2.90±1.28
Inadequate training for me on using new technologies or (LMS) for distant learning is a barrier	103 12.9)	237 (29.6)	152 (19.0)	223 (27.9)	85 (10.6)	3.06±1.23
Lacking personal interest and motivation (negative attitude) to online learning is a barrier for me.	186 (23.3)	225 (28.1)	123 (15.4)	174 (21.8)	92 (11.5)	3.30±1.34
Some disciplines or contents are not suitable for E-learning (as clinical teaching).	357 (44.6)	230 (28.7)	126 (15.8)	60 (7.5)	27 (3.4)	4.04±1.09
The most challenging learning outcome for me through distance learning is the learning skills.	176 (22.0)	329 (41.1)	173 (21.6)	79 (9.9)	43 (5.4)	3.65±1.09
Lack of fairness in student rating is a barrier to me during E-learning exams.	279 (34.9)	193 (24.1)	169 (21.1)	94 (11.8)	65 (8.1)	3.66±1.28
Limited resources such as weak internet connection and electricity shut down is a barrier to learning	453 (56.6)	197 (24.6)	87 (10.9)	48 (6.0)	15 (1.9)	4.28±1.00
The cost of accessing the internet is a barrier	236 (29.5)	179 (22.4)	170 (21.3)	162 (20.3)	53 (6.6)	3.48±1.28
The cost of buying a new device like an iPad or laptop or smartphone to help me in accessing the lecture is a barrier.	256 (32.0)	155 (19.4)	160 (20.0)	161 (20.1)	68 (8.5)	3.46±1.34

Positive ‎associations between the satisfaction level of the students were identified with female gender, fourth year of study, city center location, and house income of more than ($350, IQD 500,000) (Table 4).

**Table 4:** Differences between students’ variables with satisfaction level

Variables	Categories	Satisfaction level		P-value (Chi-square test)
		Satisfied No (%)	Not satisfied No (%)	
Age	Below and equal to 22	155(29%)	379(71%)	<0.001
	Above 22 years	127(47.7%)	139(52.3%)	
Gender	Male	102(43.8%)	131(56.2%)	0.001
	Female	180(31.7%)	387(68.3%)	
College stage	Third-year	95(28.7%)	236(71.3%)	0.001
	Fourth-year	149(42.5%)	202(57.5%)	
	Fifth year	26(36.1%)	46(63.9%)	
	Sixth year	12(26.1%)	34(73.9%)	
Place of living	City centers	151(31.9%)	322(68.1%)	0.049
	Discrete and sub-districts	108(40.9%)	156(59.1%)	
	Villages and peripheries	23(36.5%)	40(63.5%)	
Monthly family salary in Iraqi Dinar	Less than 500,000	98(40%)	147(60%)	0.108
	Between 500,000 and 1,000,000	123(34.6%)	232(65.4%)	
	More than 1,000,000	61(30.5%)	139(69.5%)	
College type	Science colleges	138(29.8%)	325(70.2%)	<0.001
	Humanity colleges	144(42.7%)	193(57.3%)	
The preferable type of electronic learning	Synchronized learning	83(51.2%)	79(48.8%)	<0.001
	Non-synchronized	64(45.1%)	78(54.9%)	
	Blended learning	104(32.1%)	220(67.9%)	
	Flipped learning	31(18%)	141(82%)	

Table 5 shows significant positive relationships between age and college type with satisfaction level regarding the E-learning (P-value<0.001) as well as gender and perceived barriers of E-learning (P-value<0.001). While the monthly family salary and the college stage were significantly negatively associated with E-learning barriers (P-value=0.015, 0.023, and 0.023, respectively). ‎

**Table 5:** Multiple linear regression for the predictor sociodemographic features for the satisfaction and perceived barriers B: Standardized regression coefficient; CI: Confidence Interval

Variables	Total satisfaction mean				Perceived barriers mean			
	B	CI		P-value	B	CI		P-value
Gender	-0.067	-0.254	0.007	0.064	0.155	0.151	0.410	<0.001
Age	0.163	0.035	0.103	<0.001	-0.024	-0.044	0.024	0.556
Place of living	0.003	-0.086	0.095	0.922	0.043	-0.034	0.146	0.223
Monthly family salary in Iraqi dinar	0.005	-0.077	0.087	0.902	-0.090	-0.181	-0.019	0.015
College type	0.086	0.020	0.270	0.023	-0.032	-0.177	0.071	0.406
College stage	0.021	-0.055	0.097	0.595	-0.089	-0.163	-0.012	0.023

## Discussion

The abrupt switch of teaching and learning to new platforms during the COVID-19 pandemic has created many challenges that educational institutions were obliged to deal with.‎ The use of technology was the only option to avoid more disruptions or a total shut-down in education. ‎Several educational platforms became popular and have replaced the “traditional” ‎classroom.‎ In this study, the most used platform to attend the online lectures was Google Meet. Google Meet and Classes were generally preferred for their effectiveness in teaching, learning, at no cost, and use on any device at any time [17]. Moreover, Google Classroom allows instructors and learners to create and manage classes, make assignments and grade the assignments online, as well as add digital materials such as YouTube videos and Google Forms to the assignments [18].

More than one-third of the participants of this study felt that using adequate supportive methods for delivering lectures (presentations, YouTube, pre-recorded videos, etc.) would aid their learning. Pre-recorded lectures are preferred [19]. However, active-learning strategies are important to consider [20].

The students did not feel that E-learning prepared them well for the exam due to poor comprehension. The same findings are supported by other studies [21]. This outcome did not seem to be balanced by the other advantages of E-learning such as ease of access to educational resources and tutor support. Technical support was found to influence the ‎perceived ease of use and usefulness, which leads to students ‎developing their experience in new technologies [22].‎

Traditional education is more costly as students have to spend money on transportation and other preparations. It also consumes time that could be used for studying or doing other learning activities. Our study has shown that most students saved time and ‎money with E-learning.‎ Al-Sammarraie et al. conducted a study among 400 Pakistani students and found that money and time are the most ‎important factors affecting campus students [23]. Saving time and cost is a considerable advantage of E-learning.

Although E-learning has significantly served the current pandemic situation at present, it faces many obstacles. The main issues were due to limited resources, such as Internet availability and quality, as well as an electrical power supply, which could be unpredictable in countries with weak infrastructure such as Iraq [24]. For some students, a lack of adequate computer skills makes it difficult to use E-learning facilities because they are unable to properly communicate with their ‎tutors to obtain the information and extra tuition that they need. These obstacles will put them at a level behind their peers. A similar barrier was identified among other students such as pharmacology students in India [25]. Instability of electricity, slow Internet connection, lack of ‎training, and preparation for the use of online learning platforms lead to students’ dissatisfaction [16].

Institutions may consider E-learning more malleable to the ‎theoretical components of the syllabus but it falls behind when it comes to courses with practical ‎components. Medical and dentistry students of Queen’s University Belfast indicated that E-learning in a practical set-up is useful [26]. However, ‎this did not apply to all their students, as those students who preferred a more superficial ‎approach to learning, like using online checklists, did not perform as well in real-time ‎when attending Objective Structured Clinical Examination OSCEs [26]. In measuring total ‎satisfaction means, only 35.25% were satisfied with their experience. A study of the ‎implementation of E-learning environments in higher education in Sweden and Lithuania ‎found that course content and curriculum type are significantly associated with the ‎level of acceptance [27].‎

Blended learning might be an optimal educational approach to use. A research ‎group in Turkey has found that most students preferred blended learning [28]. In Zambia, a ‎high acceptance rate of blended learning (>75%) was found. The flexibility in the educational style, the easier access to learning resources in times of limited internet access or electrical power, and the opportunities to have ‎face-to-face encounters in combination with streamlined feedback provision constitute the ‎main reasons why students prefer blended learning compared to fully face-to-face or ‎online education [29-30]. ‎

Our study had limitations that limited the generalization of its results to all students or colleges in Iraq. The small sample size, the general limitation of the survey questionnaire, and the lack of prior data or studies to build on are a few of the main limitations. However, results can be utilized to inform educators and institutions when structuring curricula and designing learning activities. Conducting another research that is based on the teachers' perspective besides the students' perspective is recommended.

## Conclusions

Although E-learning was the only choice for teaching and learning during the COVID-19 ‎pandemic, the absolute dependence on E-learning was challenging. Most of the students at the selected universities were not satisfied with the E-learning process. Power failures and slow Internet speed were the main barriers.

We recommend enhanced training for tutors and students on ‎online learning platforms and blended learning in order to overcome the ‎problems. Universities should invest in, and incrementally introduce, blended learning even after the end ‎of the pandemic to support traditional learning and communication. Larger and diverse study samples from other universities are recommended for future research to draw accurate ‎conclusions and construct recommendations for advancing education.
